# The Prevalence of Weight Gain After Obtaining Employment: A Cross-Sectional Survey of Employees at the Ministry of National Guard Health Affairs, Eastern Region, Saudi Arabia

**DOI:** 10.7759/cureus.56572

**Published:** 2024-03-20

**Authors:** Seham Khashwayn, Maha B Alqahtani, Saffanah A Al Katheer, Arwa A AL Hussaini, Mohammed A Bakhashwayn, Ali A Al Qarni

**Affiliations:** 1 Research, King Abdullah International Medical Research Center, Alhasa, SAU; 2 Research, King Saud Bin Abdulaziz University for Health Sciences, Alhasa, SAU; 3 College of Clinical Laboratory Sciences, King Saud Bin Abdulaziz University for Health Sciences, Jeddah, SAU; 4 Biomedical Research Department, King Abdullah International Medical Research Center, Jeddah, SAU; 5 Pathology and Laboratory Medicine, Ministry of National Guard Health Affairs, Jeddah, SAU; 6 Public Health, Ministry of Health, Dammam, SAU; 7 Research, Ministry of National Guard Health Affairs, Alhasa, SAU

**Keywords:** employees, healthcare, workplace, obesity, cross-sectional studies, weight gain

## Abstract

Background and objective

Even though obesity is a major global health concern, few studies in the literature have discussed obesity in the workplace. In light of this, we aimed to explore obesity in the workplace and its contributing factors.

Methodology

An online survey was distributed via official work emails. The survey assessed demographic variables and work environment-related factors, such as physical and mental well-being, lack of time, and social and personal habits. The total sample included 380 full-time employees, of which 16.67% were excluded for not meeting the inclusion criteria. Data were analyzed by using descriptive and inferential statistics.

Results

Of note, 79.87% of the participants reported an increase in weight after getting employed. The average weight gain was 10.4 kg for 8.2 years of experience. The physical and mental items and time-related items had the highest average scores of 3.24 and 3.44, respectively. The multivariable logistic regression showed a significant association between work experience (p = 0.0259) and time (p = 0.0363), as well as physical and mental domains (p = 0.0007).

Conclusions

Based on our findings, greater work experience, a lack of time, and negative mental and physical well-being are risk factors for weight gain among employees.

## Introduction

Obesity is defined as excessive body fat accumulation in an individual. A person with a body mass index (BMI) exceeding 30 kg/m^2 ^is considered obese [1]. In 2016, the global prevalence of obesity was estimated to be 13%, and it continues to be on the rise [2]. Obesity negatively impacts global health and the economy significantly [2,3]. Although the incidence of obesity in Saudi Arabia has been analyzed, few studies have investigated the prevalence of obesity in the workplace [4]. One study in Saudi Arabia aimed to assess the obesity prevalence among Saudi Aramco employees and reported that out of 1,000 employees, 22.5% were obese, and 36.7% were overweight [5]. With the country experiencing rapid economic growth and low unemployment rates, the impact of work on individuals’ physical activity and eating habits might contribute to an increased risk of obesity in the Saudi population [6].

Several studies have reported that obesity negatively impacts work productivity and might increase direct medical costs and absenteeism [7-10]. A study conducted in the United States (US) investigating the relationship between obesity and absenteeism in a sample of 10,825 employees revealed that employees with obesity were twice as likely to have a high level of absenteeism [11]. A recent study in Saudi Arabia evaluated the economic burden of obesity, and reported estimated costs of $3.8 billion and 15.5 billion due to direct medical expenses and absenteeism, respectively [12].

To investigate the prevalence of weight gain associated with the workplace, CareerBuilder, a US-based multinational company, conducted an online survey involving 3,420 full-time employees to assess the number of workers who reported weight gain at their current jobs. Overall, 56% of the surveyed employees reported weight gain, with 51% agreeing that this was primarily due to sitting at a desk all day [13]. Various lifestyle patterns employees adopt, such as skipping meals, increasing the number of snacks, or not participating in exercise or leisure activities, may negatively impact their health. Improper dietary habits and sedentary lifestyles have been extensively discussed in the literature and are considered risk factors for obesity. These factors are particularly relevant for employees who work 8-12 hours a day [14-18].

In light of the findings mentioned above from various studies, it is necessary to address the following question: does the work environment in any way contribute to the prevalence of weight gain and obesity among employees in Saudi Arabia? To that end, this study aims to shed light on employees' perspectives on gaining weight after getting employed in their organization. In addition, we explore any potential weight gain-associated factors related to work such as work environment, experience, position, and shift hours, among others.

## Materials and methods

Study design, participants, and setting

This cross-sectional study was conducted at the Ministry of National Guard Health Affairs (MNGHA) facilities in the cities of Alhasa and Dammam, Saudi Arabia. MNGHA is a governmental healthcare organization that provides clinical, academic, and research services. The estimated number of MNGHA employees in both the cities was 4,142. To be eligible for participation, individuals had to be Saudi or non-Saudi employees of the MNGHA-ER, aged 18 or older, and able to comprehend Arabic and/or English. Healthcare providers and non-healthcare providers of both genders were deemed eligible to participate in the study. Employees who had undergone weight loss surgery, taken weight reduction medication, or could not understand Arabic or English were excluded.

Study duration and data collection tool

Data were collected via a survey between January and the end of February 2023. The survey questions were adapted from a study conducted by CareerBuilder [13]. The questions were reformulated (Appendix A), examined in a pilot study, and reviewed by two epidemiologists before the initiation of the study. A pilot study was conducted with 10 employees to ensure the questions were comprehensible and clear. Cronbach’s alpha was calculated to evaluate the reliability of the instrument, which demonstrated a result of 0.78. The data collected during the pilot study were not included in the final dataset. Participants were asked for their consent to participate in the study before they could access the survey. Participants evaluated the degree to which the survey questions resonated with them, on a 5-point scale (5 = high resonance and 1 = low resonance). The scale used for questions 1 and 5 in the time factor domain was reversed to accommodate the negative aspects of the other survey questions.

The survey consisted of two parts. The first part comprised sociodemographic-centric questions assessing experience, age, weight, height, gender, status, occupation, work hours, education level, and the prevalence of weight gain. The prevalence of weight gain was assessed by asking the participant to recall their weight before employment and to report their current weight. The second part consisted of three domains. The first domain assessed the impact of social factors and included items related to eating due to stress, regularly ordering restaurant food to the workplace, involvement in workplace celebrations with food or high-sugar snacks, pressure to eat food brought in by co-workers, and department-provided snacks or open snacks table. The second domain assessed the impact of the time factor and included items on the absence of leisure activities, lack of time to exercise before or after work, skipping meals due to lack of time, extra shift hours, and not having time to prepare meals to eat at work. The third domain assessed physical and mental factors and examined participants’ negative feelings (mood swings, anxiety, or depression), perception of their appearance, whether they sat at a desk for most of the day, and whether they could not exercise due to work-related fatigue.

Sampling strategy and sample size calculation

The convenience sampling technique was used in the study. Employees were contacted through their office emails and were invited to participate in the study on a voluntary basis. The required sample size was calculated using the Raosoft Sample Size Calculator [19]. Based on the total employee population of 4,142, a confidence level of 95%, and a margin of error of 5%, the required sample size was 352 employees.

Data analysis and management

Data were entered into an Excel file and analyzed using SAS 9.4. Descriptive statistics were used to summarize the data. Means ± standard deviation (SD) were used for continuous numerical variables that had normal distributions. For non-normally distributed continuous numerical variables, medians and interquartile ranges (IQRs) were used. Frequencies and percentages were used to summarize categorical variables. Simple and multivariable logistic regression models were built to test the effect of survey domains and demographics on employee weight changes. The data were stored securely to maintain confidentiality.

Ethical approval

The Institutional Review Board (IRB) of the King Abdullah International Medical Research Center (KAIMRC) approved the study on December 04, 2022 (approval number: NRA22A/040/11).

## Results

A total of 380 employees responded to our survey. Less than a quarter (16.31%) of them were eliminated due to not meeting the inclusion criteria. The excluded participants were on weight loss medication (12.5%) or had undergone weight loss surgery (4.26%). Hence, the total number of participants included in the study was 318. Most participants were male (51.56%) and the average age was 36 ± 8.1 years. The majority were married (67.19%), had regular work schedules (74.38%), and were non-healthcare providers (59.12%). Less than half engaged in regular physical movement in their work (47.19%), and most did not have a chronic illness (79.06%). Most participants reported an increase in weight since they were hired (79.87%) and very few reported no change in weight (3.77%); however, some reported losing weight (16.35%; Table 1).

**Table 1:** Demographic Characteristics

Variable	N (%)
Gender	
Male	165 (51.56%)
Female	155 (48.44%)
Marital Status	
Married	215 (67.19%)
Single	105 (32.91%)
Occupation	
Non-Healthcare Provider	188 (59.12%)
Healthcare Provider	130 (40.88%)
Work Nature (i.e., Requires Regular Physical Movement)	
Yes	151 (47.19%)
No	108 (33.75%)
Sometimes	61 (19.06%)
Work Schedule	
Regular	238 (74.38%)
Shifts	82 (25.63%)
Chronic Illness	
Yes	67 (20.94%)
No	253 (79.06%)
Reported Increase in Weight	
Yes	254 (79.87%)
No	64 (20.87%)
Weight Change	
Weight Loss	52 (16.35%)
Weight Gain	254 (79.87%)
Weight Maintained	12 (3.77%)

The average weight of the respondents at the time of their hiring was 69 kg (95% CI: 67.3-70.8), while the average weight at the time of the survey was 76 ± 17 kg. The average work experience was eight years (95% CI: 7.5-8.8; Table 2).

**Table 2:** Demographic Numerical Characteristics and Calculated BMI BMI: Body Mass Index; CI: Confidence Interval; IQR: Interquartile Range; SD: Standard Deviation

Variable	Median (IQR)	Mean ± SD	95% CI
Age	35 (10)	36 ± 8.1	(26.9–35.14)
Height	166 (14)	165.9 ± 10.9	(164.7–167.1)
Reported Weight Before Hiring (kg)	68 (20)	69 ± 15.7	(67.3–70.8)
Body Mass Index (BMI)	24 (5.4)	24.8 ± 4.4	(24.3–25.3)
Reported Weight at the Time of the Survey (kg)	73 (23)	76 ± 17	(74.2–77.9)
Body Mass Index (BMI)	26.7 (7.2)	27.4 ± 5.08	(26.8–27.9)
Weight Change			
Weight Loss	4.5 (7)	7.9 ± 10.2	(5.13–10.8)
Weight Gain	8 (9.5)	10.4 ± 8.2	(9.3–11.4)
Work Experience (Years)	8 (9)	8.2 ± 5.8	(7.5–8.8)

Among the factors that influenced weight in the work environment assessed, time-related ones had the highest mean score (mean = 3.44; 95% CI: 3.35-3.54) while social and personal habits had the lowest (mean = 2.44; 95% CI: 2.34-2.52). The physical and mental factor-related items that resonated the most with employees were those related to being unable to exercise due to work-related fatigue (mean = 3.9; 95% CI: 3.7-4) and sitting at a desk for most of the day (mean = 3.4; 95% CI: 3.2-3.6). For the time factor, the items that resonated the most were having no time to exercise, no time for meal preparation, no time for leisure activity, and skipping meals, with mean scores of 3.8, 3.7, 3.5, and 3.4. respectively. In the social and personal habits realm, the items that resonated the most were regularly ordering food from restaurants to the workplace (mean = 3.1; 95% CI: 2.9-3.3) and overeating due to stress at work (mean = 2.8; 95% CI: 2.7-3.0; Table 3).

**Table 3:** Survey Items CI: Confidence Interval; IQR: Interquartile Range; SD: Standard Deviation

Variable	Median (IQR)	Mean ± STD	95% CI
Average Physical and Mental Factor Effect	3.3 (1.5 )	3.2 ± 0.9	(3.1–3.3)
Often have negative feelings, mood swings, anxiety, or depression	3 (2)	2.7 ± 1.2	(2.6–2.9)
Dislike your physical appearance	3 (3)	2.8 ± 1.4	(2.6–2.9)
Sit at a desk most of the day	4 (3)	3.4 ± 1.4	(3.2–3.6)
Cannot exercise due to work-related fatigue	5 (2)	3.9 ± 1.3	(3.7–4.0)
Average Time Factor Effect	3.4 (1.2)	3.4 ± 0.8	(3.4–3.5)
Having time available for leisure activities	4 (2)	3.5 ± 1.3	(3.4–3.6)
No time to exercise before or after work	4.5 (2)	3.8 ± 1.3	(3.7–4.0)
Skipping meals because of time constraints	4 (2)	3.4 ± 1.3	(3.3–3.6)
Often involved in extra shift hours	2 (3)	2.5 ± 1.5	(2.3–2.7)
Having time for meal preparation to eat at work	4 (2)	3.7 ± 1.4	(3.6–3.9)
Average Social and Personal Habits Factor Effect	2.4 (1.2)	2.4 ± 1.2	(2.3–2.5)
Overeating due to stress at work	3 (3)	2.8 ± 1.4	(2.7–-3.0)
Ordering restaurant food to the workplace regularly	3 (4)	3.1 ± 1.5	(2.9–3.3)
Involved in celebrations at the workplace that have food/high-sugar snacks	2 (2)	2.1 ± 1.2	(1.9–2.2)
Pressure to eat food co-workers bring in	2 (2)	2.3 ± 1.3	(2.1–2.4)
The department has snacks or an open snack table	1 (1)	1.7 ± 1.3	(1.5–1.8)

Demographic variables such as age, height, gender, marital status, occupation, physical movement, work schedule, and chronic illness history, as well as the time factor, were not associated with weight gain. However, the physical and mental factor was significantly associated with weight gain (p = 0.0016). The likelihood of being negatively affected both physically and mentally in the work environment was 63.1% more in participants who reported weight gain (Table 4).

**Table 4:** Simple Logistic Regression CI: Confidence Interval

Variable	Estimate	Odds Ratio (95% CI)	P-value
				Age	−0.00173	0.998 (0.965–1.033)	0.9208
Height	0.0177	1.018 (0.994–1.042)	0.1465
Gender (Male vs. Female)	0.0785	1.170 (0.676–2.025)	0.5747
Marital Status (Married vs. Single)	0.208	1.516 (0.861–2.667)	0.1492
Occupation (Healthcare staff vs. Admin and Faculty)	0.1533	1.359 (0.783–2.359)	0.276
Work Nature Involves Physical Movement			
Yes vs. No	−0.1744	0.588 (0.306–1.132)	0.3539
Sometimes vs. No	−0.1819	0.584 (0.263–1.298)	0.4321
Work Experience (Years)	0.0488	1.050 (0.999–1.104)	0.055
Work Schedule (Regular vs. shifts)	0.025	1.052 (0.565–1.959)	0.8738
Chronic Illness (Yes vs. No)	−0.1414	0.754 (0.396–1.435)	0.3892
Level of Effect of Physical and Mental Factors	0.489	1.631 (1.205–2.208)	0.0016
Level of Effect of Time Factor	−0.0586	0.943 (0.678–1.313)	0.7285
Level of Effect of Social and Personal Habits Factor	0.3246	1.384 (0.978–1.957)	0.0665

The multivariable logistic regression model revealed a statistically significant association between weight gain and the physical and mental factors, time factor, and work experience after controlling for the social and personal habits factor. The odds of being affected physically and mentally were 85.5% higher in those who reported weight gain. Participants who gained weight reported a lower negative effect of the time factor compared to those who did not gain weight. Moreover, those who gained weight had more work experience (Table 5).

**Table 5:** Odds Ratio Estimates CI: Confidence Interval

Effect	Estimate	Point Estimate	95% CI		P-value
Level of Effect for Physical and Mental Factors	0.6179	1.855	1.296	2.656	0.0007
Level of Effect for Time Factor	−0.4098	0.664	0.452	0.974	0.0363
Level of Effect for Social and Personal Habits	0.2981	1.347	0.929	1.954	0.1159
Work Experience	0.0580	1.060	1.007	1.115	0.0259

## Discussion

Obesity is a complex health issue with various root causes [20-35]. In this study, we assessed weight change in employees after gaining employment and explored the work environment factors that were identified as factors predisposing individuals to weight gain in previous studies [13-30]. Three main factors were employed to assess the contribution of work to obesity: physical activity, mental health, and social and personal habits. Most participants (79%) reported an average weight gain of 10.4 ± 8.2 kg during 8.2 ± 5.8 years of work experience with no obesity observed in this study participants. Similar findings were reported in a cohort study with a follow-up period of 28 years, where 64% of the sample reported weight gain (37% for males and 27% for females), with an average weight gain of 3.3 kg and 2.7 kg over 10 years for males and females, respectively [20].

Our multivariable logistic regression model showed a statistically significant association between employment duration and weight gain (p = 0.0259). The process of gaining weight can be slow and steady. Participants in this study gained an average of 10 kg in eight years. This is similar to the weight gain reported in a 37-year-long follow-up study in the US, where participants gained an average of 11 kg [21]. A previous study reported a significant BMI increase after 10 years, with an average weight gain of 3 kg [17]. In our study, we found no significant association between age and weight. This could be attributed to weight increasing along with age [31].

In terms of survey outcomes, the physical and mental factor domain covered items such as having negative feelings (mood swings, anxiety, or depression), disliking one’s appearance, sitting at a desk most of the day, and being unable to exercise due to work-related fatigue. A higher effect of this domain was observed among participants who reported weight gain. The items that resonated the most were related to being unable to exercise due to work-related fatigue (3.9 ± 1.3) and sitting at a desk for most of the day (3.4 ± 1.4). Several items showed low resonance, including disliking one’s appearance (2.8 ± 1.4), while a neutral level of resonance was seen for the item related to having negative feelings (2.7 ± 1.2). Similarly, another study found that high BMIs were observed in individuals who spent a longer time sitting at their desks [22]. An experimental study examined if employees sit more at work than in their leisure time and confirmed that employees spend less time sitting during leisure time [23]. Thus, being active in the workplace should be encouraged, as a movement as simple as stair-climbing can improve body composition [24].

The time factors assessed in this study included being unavailable for leisure activities, having no time to exercise before or after work, skipping meals due to time constraints, picking up extra shift hours, and not having time to prepare meals to eat at work. The item that showed the highest resonance was having no time to exercise before or after work (3.8 ± 1.3), followed by not having time to prepare meals to eat at work (3.7 ± 1.4), being unavailable for leisure activities (3.5 ± 1.3), skipping meals (3.4 ± 1.3), and picking up extra shift hours (2.5 ± 1.5). Similar findings have been reported in several studies where participants who prepared meals described better food quality, lower BMIs, and better energy [25-28]. Extra shift hours and increased sedentary behavior have been linked to weight gain and obesity in the long term [29,30]. A case study in Denmark revealed that a lack of time was the most frequently cited reason for the decrease in the time spent with family, a lack of physical activity, and a lack of leisure activity. However, the researchers concluded that time is not a fixed factor and relies on the negotiation, priorities, and responsibilities of an individual [32].

To the best of our knowledge, this study is the first of its kind to be conducted in Saudi Arabia. In the context of our fight against obesity, the current findings highlight the important role of factors that predispose employees to obesity that could be modified. However, as an observational study, it has a few limitations, as the data collected was self-reported and, therefore, is subject to recall bias (such as for weight recall). In addition, many factors known to affect weight were not assessed, such as smoking status, sleeping hours, family size, and income, which may have confounded the results. Furthermore, the low response rate limits the generalizability of the findings, as does the fact that the study was conducted at a single organization. We recommend conducting more studies on the topic by drawing on the premise and approach of this study at several organizations in Saudi Arabia, albeit with a more advanced study design, such as a cohort study.

## Conclusions

Our study explored a rarely discussed factor related to obesity in Saudi Arabia. The prevalence of weight gain after obtaining employment was reported to be 79% among MNGHA-ER employees. Three main factors were found to contribute to weight gain in the workplace: work experience, a lack of time, and physical and mental factors. This highlights the need for the implementation of obesity prevention, time management, and mental health interventions in the workplace.

## Appendices

Appendix A:

Inclusion and exclusion criteria questions:

Do you want to participate in the study? 1) Yes. 2) No

Have you ever taken weight reduction medication while working at MNGHA? 1) Yes. 2) No

Have you undergone any weight loss surgery? 1) Yes. 2) No

**Table 6:** First Part: Demographics

Demographics	Options	Type of Variable
Age		Numerical
Have you noticed any weight gain before and after joining MNGHA?	1) Yes. 2) No	Categorical
Approximate weight before joining MNGHA (kg)		Numerical
Current weight (kg)		Numerical
Height (cm)		Numerical
Gender	1) Male. 2) Female	Categorical
Status	1) Single. 2) Married	Categorical
Occupation	1) Doctor. 2) Pharmacist. 3) Nurse. 4) Allied Health. 5) Admin. 6) KSAU-HS Faculty. 7) Other (Specify)	Categorical
Does your work nature include physical movement?	1)Yes. 2) No	Categorical
Work experience in years at the National Guard?		Numerical
Work schedule	1) Regular. 2) Shifts	Categorical
Living with chronic illness. Specify if yes	1) Yes. 2) No	Categorical
		Categorical

**Table 7:** Second Part: Survey Questions

Item	1	2	3	4	5
Often have negative feelings, mood swings, anxiety, or depression					
Dislike your physical appearance					
Sit at a desk most of the day					
Cannot exercise due to work-related fatigue					
Having the availability for leisure activities					
No time to exercise before or after work					
Skipping meals because of time constraints					
Often involved in extra shift hours					
Having time for meal preparation to eat at work					
Overeating due to stress at work					
Ordering restaurant food to the workplace regularly					
Involved in celebrations at the workplace that have food/high-sugar snacks					
Pressure to eat food co-workers bring in					
The department has snacks or an open snack table					
